# Innovative applications and therapeutic potential of oilseeds and their by‐products: An eco‐friendly and sustainable approach

**DOI:** 10.1002/fsn3.3322

**Published:** 2023-03-22

**Authors:** Ifrah Usman, Hina Saif, Ali Imran, Muhammad Afzaal, Farhan Saeed, Iqra Azam, Atka Afzal, Huda Ateeq, Fakhar Islam, Yasir Abbas Shah, Mohd Asif Shah

**Affiliations:** ^1^ Department of Food Sciences Government College University Faisalabad Faisalabad Pakistan; ^2^ Department of Food Sciences Technology Chulalongkorn University Bangkok Thailand; ^3^ Department of Food Sciences Government College Women University Faisalabad Faisalabad Pakistan; ^4^ Department of Economics, College of Business and Economics Kebri Dehar University Jigjiga Ethiopia; ^5^ Adjunct Faculty, University Centre for Research & Development Chandigarh University, Gharuan Mohali India

**Keywords:** industrial applications, nutraceutical foods, oilseed, oilseed by‐products, sustainability, therapeutic application

## Abstract

The risk of inadequate management of agro‐waste is an emerging challenge. However, the economic relevance of agro‐waste valorization is one of the key strategies to ensure sustainable development. Among the agro‐waste, oilseed waste and its by‐products are usually seen as mass waste after the extraction of oils. Oilseed by‐products especially oilseed cakes are a potential source of protein, fiber, minerals, and antioxidants. Oilseed cakes contain high value‐added bioactive compounds which have great significance among researchers to develop novel foods having therapeutic applications. Moreover, these oilseed cakes might be employed in the pharmaceutical and cosmetic industries. Thus, as a result of having desirable characteristics, oilseed by‐products can be more valuable in wide application in the food business along with the preparation of supplements. The current review highlights that plentiful wastes or by‐products from oilseeds are wasted if these underutilized materials are not properly valorized or effectively utilized. Hence, promising utilization of oilseeds and their wastes not only assists to overcome environmental concerns and protein insecurity but also helps to achieve the goals of zero waste and sustainability. Furthermore, the article also covers the production and industrial applications of oilseeds and by‐products along with the potential role of oilseed cakes and phytochemicals in the treatment of chronic diseases.

## Graphical abstract

A broad range of valuable bioactive compounds has been obtained from oilseeds either in pure form or in a homogenous mixture. Oilseed cake or meal had been undervalued by‐products or waste, which conventionally had been utilized for feed in many countries. In addition to a wide range of carbohydrates, proteins, fibers, vitamins, antioxidants, and bioactive compounds are also present in varying quantities. Oilseeds are widely used for human consumption and industrial application. To overcome this problem, they are in constant search to find ways to reutilize agro‐industrial waste or by‐products.

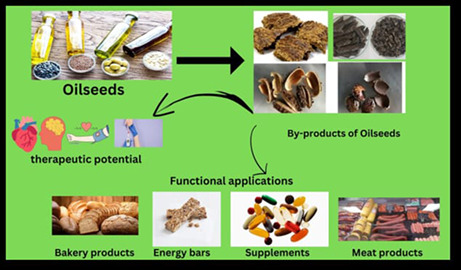

## 1 INTRODUCTION

A broad range of valuable bioactive compounds has been obtained from oilseeds either in pure form or in a homogenous mixture. Oilseed cakes or meals had been undervalued by‐products or waste, which conventionally had been utilized for feed in many countries. Oilseed meals are valuable for animal consumption because of their abundant energy, protein, vitamin and minerals, etc. Oilseeds are highly valuable plant‐based products containing an elevated level of essential oils and other food fats (Yadav, Meena, et al., 2020; Yadav, Yadav, et al., 2020). In addition to a wide range of carbohydrates, proteins, fibers, vitamins, antioxidants, and bioactive compounds are also present in varying quantities. Oilseeds are widely used for human consumption and industrial application (Yadav, Meena, et al., 2020; Yadav, Yadav, et al., 2020). These oilseeds are popular throughout the world owing to their excellent therapeutic potential. Due to unique properties, the by‐products of oilseeds have gained attention for utilization in the development of functional foods and other value‐added products, for this purpose, innovative technologies are being utilized (Usman et al., 2022). Oilseed meals or cakes are utilized as protein isolates, hydrolysates, and bioactive peptides. One of the cost‐free uses of oilseed meal is its utilization as plant compost which requires no processing. By considering all of these, entrepreneurs are interested in the commercialization of natural agro‐waste or by‐products. Due to augmented protein demand, the oilseeds generation has enormously elevated resulting in the accumulation of a huge amount of waste and by‐products from oilseeds processing. To overcome the problem, they are in constant search to find ways to reutilize such wastes or by‐products. Because of its high moisture content, agro‐waste is easily susceptible to spoilage but (Table 1) the waste from agro‐industries has the potential to be used as a renewable energy source (Babu et al., 2022). Oilseed crops are important due to their ability to support the ever‐growing biofuel industry (Lietzow, 2021). After oil extraction, increased financial encouragement can obtain by extraction of vital coproducts from oilseed meal residue. A synthetic antioxidant is helpful, contrary to this it can also have negative impacts on health, for example, potential enzyme, lipid, and pathological alterations. As a result, the utilization of natural antioxidant from plants especially oil meal especially mustard and sesame oilseed meals have gained attractiveness (Saeed et al., 2022). The different seeds meal has been utilized for a reduction in lipid oxidation, to preserve quality, and to extend shelf life in meat. The vital oilseed crops can be utilized separately as well as incorporated in various food products as a key ingredient. Bioactive lipids from different oilseeds have conversed with the protection, enhanced oral delivery, and controlled release when synthesized into different colloidal systems such as emulsion, oleofoams, and oleogels. These have been widely used for the improvement of sensorial, technological, and fortification purposes (Lee et al., 2022). Furthermore, seeds are also reported to reduce the diseases like atherosclerosis, oxidative stress, and cardiovascular, etc. (Jayaraj et al., 2020). The evidence showed that these types of biological effects are because of a certain type of lignin. Several oilseeds are loaded with this lignin. Furthermore, seeds have various phenolic compounds, such as ferulic, cinnamic p‐coumaric, and vanillic acids. Similarly, oilseeds have been utilized for the treatment of various ailments such as arthritis and rheumatism, for aching feet as a foot bath, and in a plaster form, to cure pneumonia and bronchitis on the chest and back (Mayekar et al., 2021). Some oilseeds are loaded with selenium and magnesium, due to which it has anti‐inflammatory characteristics. Moreover, it proved to help activate sweat glands and reduce body temperature. In conventional medicine, it is utilized to relieve the pain due to arthritis, muscle strain, and sprain (Mayekar et al., 2021). A few oils are not merely edible oil it is also well‐known medicine in the health care Ayurveda system. For example, mustard is also used for joint and muscular problems and therapeutic massages. A few oilseeds in combination with turmeric and garlic are utilized for joint pains and rheumatism. In addition to this utilized for mosquito repellent, oilseed meals or cakes are utilized as protein isolates, hydrolysates, and bioactive peptides. This review summarizes the functional applications and therapeutic applications of oilseeds and their by‐products obtained during processing.

**TABLE 1:** Chemical composition of different oilseed meals.

	Canola	Sunflower	Chia	Olive	Coconut	Rapeseed	Soybean	Walnut	Sesame	References
Moisture %	7.3–12	2.56–10	10.47	N.a	5.8–9.9	3.96–10.6	88.7	10.6	4–10	Jannathulla et al. (2018)
Dry matter %	87.91–92.7	90–97.4	89.53	N.a	90.1–94.3	88–96.4	11.3	89.5	90–96	Tofanica and Gavrilescu (2019)
Crude protein%	34.5–40	31.9–43.38	41.36	0.3–10.6	17.8–20	16–45	46–52.4	13–38.9	22.65–48.5	Oliveira et al. (2017)
Ash %	4.19–6.34	6.4–7.83	7.24	3.4–9.1	2.02–4.9	6.1–15.8	2–6.96	7.48	5.3–13	Capitani et al. (2012)
Crude fiber %	12–20.9	13.07–28	27.57	N.a	10.3	8.2–17.5	4.4–75	5.33	4–10.36	Zheng and Li (2018)
Crude lipid %	2.5–19.4	1–23.6	0.21	4	3.22–9.4	1.1–10	0.55–8	2.45–10	1.07–30	del Mar Contreras et al. (2020)
Carbohydrate %	8.35–17	25.99	23.62	N.a	47.7	21.4–47.8	43–47	12.65	25.5–34	Pop et al. (2020)

## 2 OILSEEDS

Oilseeds are nutrient dense and can be a superior fragment of the human diet. They mark a significant source of vegetable oils. Oilseeds are a good source of various macronutrients (carbohydrates, fat, protein, and fiber) and micronutrients (vitamins, minerals, and phytochemicals; Yang et al., 2019). Peanut, soybean, sesame seed, chia seed, hemp seed, and kenaf seeds are some important examples of oilseeds and contain essential micronutrients for the normal functioning of the nervous and digestive system. The presence of phytochemicals helps in reducing inflammation and boosting immunity as they retain antioxidant activity, while the minerals of oilseeds can help in enzymatic and metabolic processes (Ancuța & Sonia, 2020).

## 3 PROCESSING OF OILSEED AND OILSEED MEAL PRODUCTION

The processing of oilseeds involves the following steps cleaning, treatment, drying, dehulling, size reduction, flaking, and oil extraction (Li et al., 2022). Before processing oilseeds, it is necessary to clean them to remove unwanted foreign material, i.e., leaves, sticks, stones, metal, dirt, stem, thread, etc. To remove antinutritional compounds, it is essential to give prior treatments like washing, cooking, germination, microwaving, ultrasound, etc. After moisture reduction by drying, it is necessary to lose the hull for its easy removal from the seed. Table 2 shows the antinutritional factors of different oilseeds.

**TABLE 2:** Antinutritional factors.

Oilcake	Effect	References
Phytic acid		
Hempseed, soybean, groundnut	Reduce starch and protein absorption, promotes mineral unavailability	Pojić et al. (2015)
Tannis		
Rapeseed, hempseed, sesame, sunflower	Leads to imbalance in production of amino acid, inhibits normal protein adsorption	Jannathulla et al. (2018)
Sinapine		
Rapeseed/Canola	Off‐taste, compromised nutritional profile, induces dark color	Tan et al. (2011)
Chlorogenic acid		
Sunflower	Affects the organoleptic profile, changes the product stability and storage life	Stodolak et al. (2020)
Glucosinolates		
Hempseed, rapeseed	Promotes liver enlargement, negatively alters the regular functionality of thyroid	Pojić et al. (2015)

Oilseed seed processing produces oilseed meals as a by‐product (Yong & Wu, 2022). According to a survey, oilseed production in 2019/2022 increased to 580.69 million metric tons which in return produced a huge amount of oilseed meals. But this by‐product is quite beneficial and can be supplemented into the diet of the undernourished community in the form of food products and supplements (Ancuța & Sonia, 2020).

## 4 EDIBLE OILSEED CAKES/MEALS

Oilseed meals are seed flours that have had their fat content entirely reduced or removed; hence, the resulting seed meals have a higher protein content. As part of a waste management system, it is highly desirable to use oilseed meals as a source of functional ingredients that can be incorporated into various foods (Kotecka‐Majchrzak et al., 2020). Oilseed by‐products, including meals from soybean, peanuts, hemp seed, sesame seed, chia seed, and kenaf seeds, are low in cost but are highly nutritious, causing them to gain much attention as alternative ingredients for various food products and dietary supplements for human health benefits.

## 5 INNOVATIVE OILSEED MEAL APPLICATION IN THE PRODUCT DEVELOPMENT SECTOR

Functional foods have been developed for almost all food categories, including dairy, confectionery, beverage, bakery, and baby food markets (Siró et al., 2008). Furthermore, researchers are increasingly interested in using underutilized oilseed meals as functional ingredients in food products.

### 5.1 Bakery products

Among other functional foods, bakery items are supposed to offer the greatest variety of gluten‐free options. For this reason, these products including cakes, cookies, and bread can be supplemented with edible oilseed meals that are high in protein and fiber (Bochkarev et al., 2016). Meals from these seeds are said to be a good source incorporated into the diet for the undernourished community supplements (Ancuța & Sonia, 2020). As one of the well‐known bakery treats, cookies are regarded as a healthy means of incorporating nutrients into the diet. In a study, oilseed meals including poppy, sesame, chia, and flaxseeds have been used to replace the usual wheat flour of cookies recipe. This expands the nutritional profile while introducing innovation (Martínez et al., 2021).

### 5.2 Meat products

Oilseed proteins and fibers have been practiced to replace meat with plant‐based ingredients for enrichment purposes. Burgers have already been a point of interest for some researchers who experimented with chia seed meal enrichment. Porcine fat and beef meat mixture in hamburgers can be partially replaced with textured soy proteins (TSP) and partially defatted chia flour (PDCF), according to the research by Souza et al. (2015). The resulting product with TSP and PDFC showed improved product quality than before. In parallel with research by Rabadán et al. (2021) chia and poppy seeds were successfully incorporated into burgers resulting in low‐calorie and high carb products. They proposed that seed oils and flours may be used to create burgers with superior health advantages, comparable physical qualities, and palatability to standard burgers.

### 5.3 Beverage products

Oilseed meals' large spectrum of health value and practical qualities have received much consideration in their possible use as supplemental ingredients in a variety of foods, like beverages. Łopusiewicz et al. (2019) have investigated flaxseed meal‐based fermented beverages in comparison with kefir as substrate. Successful inoculation of kefir grains was done with three different variations that contained (flaxseed meals) with varying concentrations of 5%, 10%, and 15% w/w.

Enzymatic hydrolysis was used by Pap et al. (2020) to valorize hemp seed meal, formulating two fractions, liquid as well as sediments having various nutritive qualities, antinutrient contents, and sensory traits. For diverse product formulation, better potential has been observed by sediment fraction as compared to liquid, since it contained both ample and minor nutrients. To enhance the nutritional properties of liquid foods such as smoothies and drinks etc., the valorization of liquid fraction is permitted as a supplement.

### 5.4 Other types of products

New products always focus on the ingredients that either is novel but have never been used or the by‐products that have been valorized (Ancuța & Sonia, 2020). To guarantee the successful implementation of these ingredients, a suitable marketing strategy should be created. For example, a functional food bearing the health significance of its components can be attractive to customers.

#### 5.4.1 Energy bars

Energy bars were made by Norajit et al. (2011), using extruded combinations of rice flour supplemented at different amounts (0%, 20%, 30%, and 40%) with whole hemp seed powder and hemp seed meals. The effect of hemp powder and its level was studied against the physicochemical and antioxidant qualities of the fortified foodstuffs. Table 3 shows the antioxidants present in oil cakes.

**TABLE 3:** Various antioxidants in oil cakes.

Antioxidants			Oilcakes			References
Canola						
Gallic acid	Catechin	Luteolin	Caffeic acid	Quercetin	Ferulic acid	Şahin and Elhussein (2018)
Rapeseed						
P‐coumaric	Catechin	Caffeic acid	Epicatechin	Ferulic acid	Sinapic acid	Teh and Bekhit (2015)
Sunflower						
Chlorogenic acid	Catechin	Epicatechin	p‐coumaric			Şahin and Elhussein (2018)
Olive						
P‐coumaric	Luteolin	P‐hydroxybenzoic acid	Lignans	Quercetin		Sarkis et al. (2014)
Linseed						
Tannic acid	Ferulic acid	P‐hydroxybenzoic acid	Lignans	p‐coumaric		Şahin and Elhussein (2018)
Hemp						
Caffeic acid	Quercetin					Teh and Bekhit (2015)
Mustard						
Sinapic acid	Ferulic acid	Caffeic acid	p‐coumaric			Senanayake et al. (2019), Aider and Barbana (2011)
Sesame						
p‐coumaric	Ferulic acid	Lignans				Senanayake et al. (2019), Sarkis et al. (2014)
Peanut						
p‐coumaric	Caffeic acid					Teh and Bekhit (2015)
Palm						
p‐coumaric	Caffeic acid	P‐hydroxybenzoic acid				Senanayake et al. (2019)
Flaxseed						
p‐coumaric	Ferulic acid					Şahin and Elhussein (2018)
Cottonseed						
Ferulic acid	Quercetin					Şahin and Elhussein (2018), Teh and Bekhit (2015)

#### 5.4.2 Functional supplements

**FIGURE 1:**
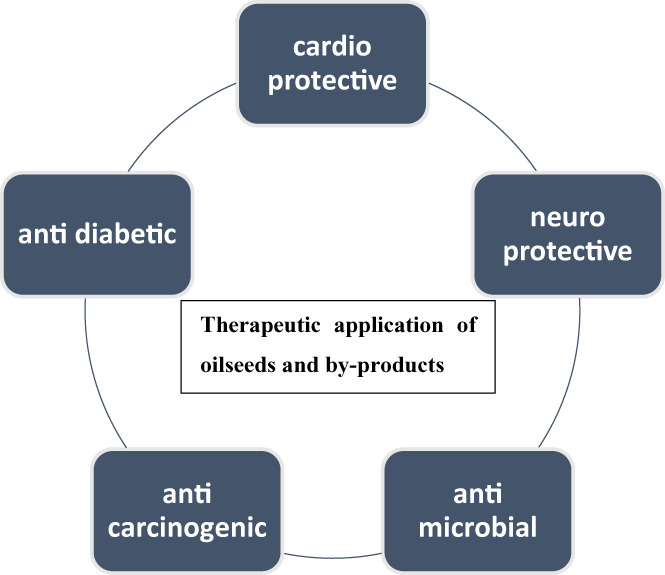
Therapeutic application of oilseeds and by‐products.

Recently, compressed tablets from four oilseed meals, such as sunflower, coconut, pumpkin, and flax, were prepared that can be utilized as useful supplements (Sobczak et al., 2020). Starch coatings were applied to enhance the hardness and cutting resistance of tablets. Moreover, enrichment was done by honey powder while caramel and chocolate coatings made them ready to eat the product. Figure 1 showed some therapeutic potentials of using oilseed products.

## 6 CONSUMER ACCEPTANCE OF NEWLY FORMULATED PRODUCTS

It can be difficult to reformulate new food products so that customers would accept them on a sensory level. This is especially true of products that contain elements from oilseed meals. Not everyone is eager to test new items, a condition known as food neophobia, despite the upgraded nutritional significance of food (Rabadán et al., 2021). It is becoming more typical for meat products to have incorporated dietary fiber from plants (Zawawi et al., 2014). It can successfully increase the new food product's acceptance and various processing features, as well as its quality and shelf life (Talukder, 2015). Although product reformulation always focuses on consumer preference, some sensory alterations may affect the acceptability of reformulated product (Rabadán et al., 2021). This problem can be resolved to some extent by introducing Food Neophobia Scale (FNS) while developing new products or strategies (Choe & Cho, 2011). This can lead the upgraded products to be well received when offered to this particular consumer category.

## 7 BIOTECHNOLOGICAL APPLICATIONS OF OILSEEDS

**FIGURE 2:**
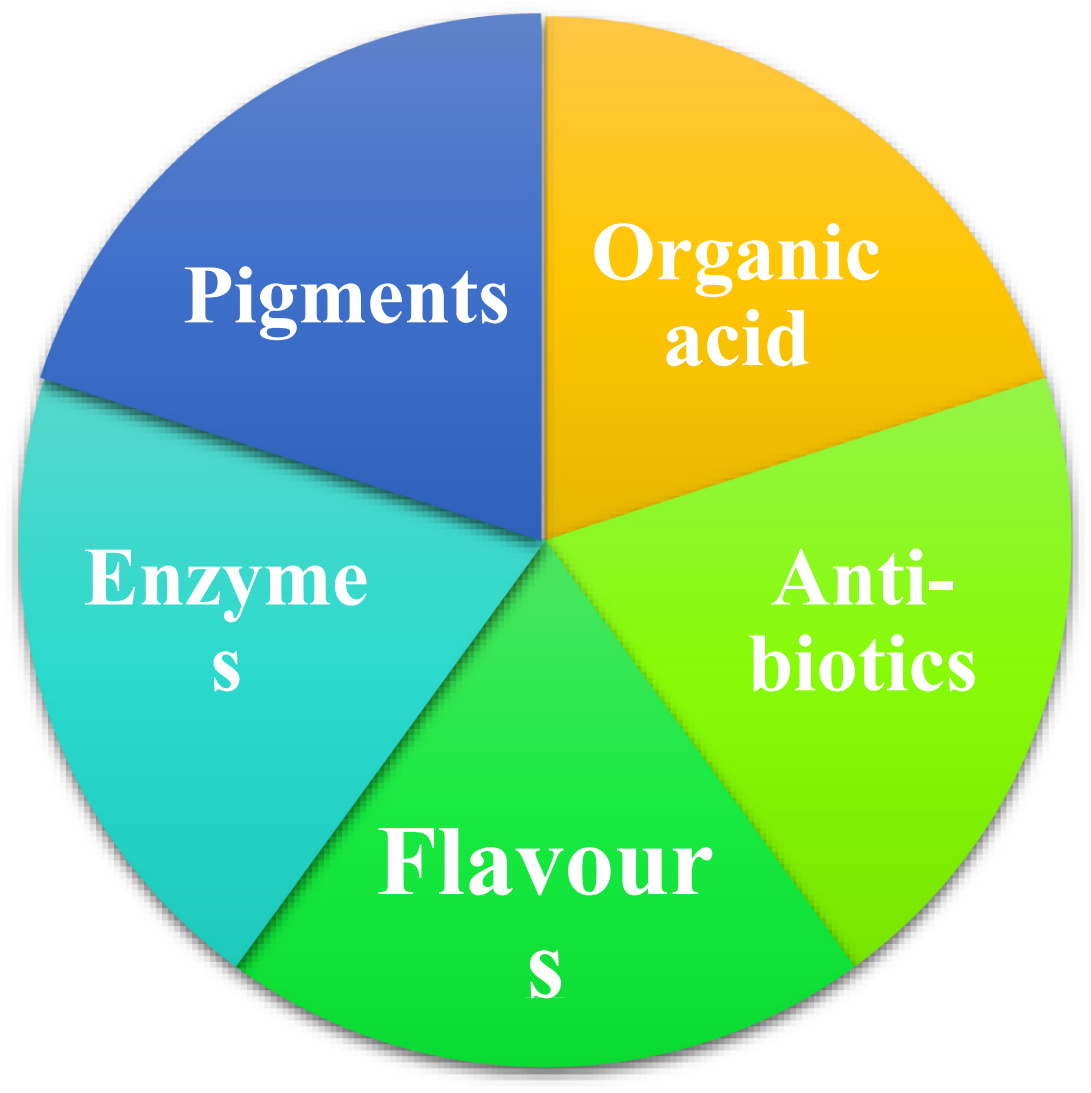
Biotechnological applications of oilseed and their by‐products.

Oilseed meals or cakes utilized for the production of organic acids, amino acids, surfactants, pigments, enzymes, single‐cell protein, flavors, mushrooms, and diet formulas for undernourished people, weaning foods, breakfast cereals, fabricated foods, multi‐purpose supplements, and many more. Some widely used applications are discussed below while Figure 2 shows the biotechnological applications of oilseed and their by‐products.

### 7.1 Production of enzymes

It was reported by many researchers that in solid‐state fermentation, oilseed cakes are used as a substrate or in the production medium as a supplement. Oilseed meals or cake are identified to be rich nutrient sources especially carbon and nitrogen for medium. For fungal species, they are highly compatible (Table 4). Among the enzymes produced using oilseed meal protease, alpha‐amylase, L‐glutaminase, phytase, inulinase, mannose, glucoamylase, and tannase are important (Chatterjee et al., 2015). In solid‐state fermentation by using fungal and bacterial strains growing on different oil cakes like soyabean oil cake, sesame oil cake, coconut oil cake, palm oil cake, and olive oil cake, protease enzyme has been produced (Gupta, Sharma, Pathak, et al., 2018; Gupta, Sharma, Sharma, & Singh, 2018). Using fungal species, for example, *Candida rugosa*, *P. simplicissimum*, *Rhizomucor pusillus*, and *P. chrysogenum*, among all enzymes, lipase has been produced earliest using sesame oil meal, coconut oil meal, olive oil meal, etc. (Treichel et al., 2010).

**TABLE 4:** Production of enzymes through solid‐state fermentation using oil cakes as substrate.

Oilcakes	Microorganisms	References
Lipase		
Olive	*Rhizomucor pusillus, Rhizopus*	Moftah et al. (2012), Thakur et al. (2015)
Sesame	*Penicillium Chrysogenum*	
Soybean	*Bacillus horikoshi*	
Coconut	*Candida rugosa*	
Cotton	*Penicillium strain*	
Phytase		
Rapeseed	*Aspergillus ficuum*	Ramachandran et al. (2007)
Coconut	*Aspergillus terreus UF39*	
Protease		
Jatropha	*Paecilomyces variotii*	Moftah et al. (2012), Gupta, Sharma, Pathak, et al. (2018); Gupta, Sharma, Sharma, and Singh (2018)
Mahua	*Aspergillus niger*	
Rapeseed	*Aspergillus oryzae*	
Olive	*Candida utilis*	
L‐Asparaginase		
Palm	*Aspergillus wentii*	Uppuluri et al. (2013)
Coconut	*Serratia marcescens*	

### 7.2 Production of mushroom

It has been reported that by solid‐state fermentation, mushrooms have been produced utilizing oilseed meal or cake. These have excellent antioxidant properties which pass during growth. Jatuwong et al. (2020) reported that when mushrooms were supplemented with cotton oilseed meal many times, increase in yield was observed. In addition to this, the obtained mushroom was found to be rich in protein and fat. The combination of different foods and oilseed meal or cake used for mushroom production and maximum increment in mushroom yield was observed as compared to the unsupplemented substrate. For the curries and soups, prepared mushrooms can be utilized (Sadh et al., 2018). Mushroom is a rich source of protein, calcium, selenium, B Vitamins, and vitamin C. Moreover, antioxidant ergothioneine is also present in it because of this property mushrooms can be consumed directly or in several foods as an ingredient.

### 7.3 Production of antibiotics and antimicrobials

Oilseed meal or cake is known to be a rich substrate for the production of antimicrobials and antibiotics (Gupta, Sharma, Pathak, et al., 2018; Gupta, Sharma, Sharma, & Singh, 2018). Sesame oil meal has been utilized for antibiotic production. As a source of carbon, sesame oil cake is utilized with phosphate buffer and during production, an intermediate is formed which is efficiently transformed into final products. Furthermore, for the production of cephamycin C and clavulanic acid, different oil cakes such as soybean meal, sunflower oil meal, and sesame oil meal have been utilized. It was reported that using sunflower oil cake, Bacitracin, a‐endotoxin produced and by using sesame oil meal *Bacillus thuringiensis* and *Bacillus licheniformis* were produced (Sarkar et al., 2021).

## 8 THERAPEUTIC POTENTIAL OF OILSEEDS AND THEIR BY‐PRODUCTS|

### 8.1 Anticarcinogenic effects

Dietary polyphenol demonstrates a double role in cancer treatment, as they have been recognized as having potential in chemoprevention as well as cure (Augustine & Bisht, 2016). Various epidemiological studies concerning chemopreventive effects suggested that intake of polyphenol‐enriched foods, as well as supplements, can minimize the development of colorectal, breast or prostate, and lung cancer (Akbari et al., 2019). Polyphenol due to their anti‐inflammatory and antioxidant properties plays a significant role as an anticancer because the tumoral environment is related to oxidative stress and inflammation (Maruca et al., 2019). Various preclinical trials have proved the positive effect of the dietary intervention of polyphenol‐enriched food on cancer appearance as well as progression. It was supported by various research pieces of evidence that supplements loaded with polyphenols display anticarcinogenic mechanisms and in vivo intracellular targets because they can regulate several enzymes and signaling pathways interlinked with oxidative stress, cellular growth, and inflammation by gene expression modulation (Ricketts & Ferguson, 2018). By vascular network formation, the polyphenols can provoke nutritional privation.

### 8.2 Antidiabetic effect

In prevention as well as treatment, dietary intervention plays a vital role in type 2 diabetes mellitus (T2D) as coadjuvants. The clinical trials with prediabetic and healthy volunteers displayed that polyphenols loaded beverages and foods greatly reduce postprandial glucose levels in the blood (Aksoylu Özbek & Günç Ergönül, 2022). This effect can be mediated by a reduction in insulin resistance. The role of *Vaccinium myrtillus* extract in bilberry consumption was reported by Smeriglio et al. (2019) that male patients having T2D observed a significant reduction in postprandial glycemia as well as insulinemia; moreover, supplementation of polyphenol acts as antidiabetic coadjuvant agent. In addition to this, it was reported that a combination of (oat) beta‐glucan, (agave) inulin, and (blueberry pomace) polyphenol food supplementation helped to improve metformin tolerance in males suffering from T2D and were intolerant to this drug. Moreover, research was done to explain the action mechanism through which polyphenol‐enriched supplements helped to improve glucose control and ameliorate the symptoms or complications of T2D (Shay et al., 2015). The cell culture assay also presented advanced information to understand the promising role played by polyphenol‐loaded food on T2D management. The potential advantage of dietary intake with food obtained from the plant on the glucose level in blood may be mediated by enhancing activated kinase protein expression level (Jugran et al., 2021). In the mice model suffering from obesity, the raspberry intake activated AMPK, which helped to enhance the expression level of GLUT‐4 glucose transporter on skeletal muscles. Hence, in diabetic patients, enhanced glucose uptake by skeletal muscle may contribute to improving glucose levels in the blood. The role of interaction between diabetes start, succession, and the gut microbiome is still weak; however, emerging evidence supports the advantages of microbial population modulation to ameliorate T2D symptoms.

### 8.3 Neuroprotective effect

In a large number of researches, the authors have suggested that the intake of polyphenols from different food sources or preparation has promising effects on central nervous system (CNS) including the improvement of cerebral blood flow, cognitive performance in cognitively impaired individuals, and prevention of the development of neurodegenerative disorders (Morya et al., 2022). The antioxidant potential of polyphenols is utilized for mental health treatment. Many studies showed the neuroprotective effects of dietary intervention with polyphenol‐enriched foods by intracellular signaling cascade modulation as well as transcription factors which help to regulate neuroinflammation and oxidative stress. Álvarez et al. (2022) reported that feeding patients suffering from stress‐mediated depression, with bioactive polyphenol‐enriched foods preparation showed better resilience. Various studies showed that polyphenol‐enriched preparation was found to be able to modulate (CBF) cerebral blood flow as well as the spatial location of the cerebrovascular network. The cerebrovascular system failure results in energy substrate shortage, cognitive malfunction, and neural integrity disruption (Zhou et al., 2019). Polyphenol‐loaded food stimulated nitric oxide (NO) production and enhanced vascular endothelial growth factor (VEGF); hence, improving angiogenesis as well as the flow of blood in postischemic neo‐vascularization rats; proving the positive potential of polyphenols, toward neuronal disorders.

### 8.4 Cardiovascular promising effects

Cardiovascular disease referred to a group of disorders that can affect the blood vessel and heart. Some of these are congestive heart failure or coronary heart disease. The risk factors for these diseases include atherosclerosis, and elevated blood pressure which can be controlled or minimized by the dietary intervention (Chaliha et al., 2020). Bioactive compounds present in oilseeds and their by‐products can be extensively utilized for the treatment as well as prevention of cardiovascular diseases (Giglio et al., 2018). Their promising effects on human health depend upon factors, i.e., amount consumed and their bioavailability. Many researchers reported that oilseeds have excellent effects on the vascular system by decreasing blood pressure, elevating antioxidant defenses, improving endothelial functions, inhibiting platelet aggregation as well as low‐density lipoproteins oxidation, and minimizing inflammatory responses.

### 8.5 Blood pressure regulator effect

The effect of polyphenolic foods might be interlinked with gender. Plant‐derived polyphenol‐enriched foods' regular consumption help to reduce both systolic and diastolic blood pressure in hypertensive female patients with polyphenol foods dietary intervention. On the other hand, significant effect against hypertension was observed in male (Suomela et al., 2016). In hypertensive patients, an improvement in endothelium function was observed with a six‐portion daily intake of polyphenol‐enriched diet from plant‐based foods reported by Noad et al. (2016). Moreover, it was observed that patients suffering from hypersensitive supplemented polyphenol might be mediated with an increment amount of active circulating endothelium progenitor cells, accountable for maintaining as well as repairing endothelium. Collectively, the results showed that dietary polyphenols encourage vasodilatation and upgrading of endothelium function, which promotes hypertension management. Furuuchi et al. (2018) observed a reduction in aortic reactive oxygen species ROS level on an increased fat diet mice model following plant‐based polyphenol consumption.

## 9 ROLE OF THE OILSEED IN SUSTAINABILITY

By 2050, the global demand for animal protein is expected to double, despite the negative environmental impacts of its production (Yadav, Meena, et al., 2020; Yadav, Yadav, et al., 2020). At the same time, there is a growing trend toward incorporating plant‐based protein into one's diet. Exploiting these underutilized materials as potential sources of ingredients can support the demand for plant protein globally (Arrutia et al., 2020). Hence, using these oilseed meals is a sustainable way to contribute to the development of low‐cost, novel, nutrient‐rich products while reducing food waste disposal. Indeed, for the oilseed industry, the demand for oilseed meals has surpassed the seed oil demand.

## 10 SUMMARY AND FUTURE PERSPECTIVES

The available studies have revealed the importance of these oilseeds' by‐products. These neglected materials are loaded with valuable proteins, dietary fibers, and bioactive compounds, according to the literature and databases that are available, hence, supporting their remarkable use as potential sources of bioactive substances for the development of innovative functional or nutraceutical products. Previously, these oilseed meals have been successfully incorporated into a variety of products such as meat, bakery, and beverage industry. Apart from its nutritional aspect, its valorization can help achieve the zero‐waste challenge. However, there is still a substantial gap since it is difficult to use oilseed meals in sustainable ways. Some of these issues include fresh meal degradation, antinutrient removal, allergic reactions, and consumer disapproval of such products.

## 11 CHALLENGES ASSOCIATED WITH OILSEED BY‐PRODUCT VALORIZATION

Oilseed meal output is rising globally, but the utilization catalog is poor, suggesting less output from plentiful assets. There are many challenges associated with oilseeds and their by‐product valorization as effective drying methods for precise oilseed meals or cakes are critical to enhancing the number of waste produced from industries, and the energy and cost consumption in these drying techniques should be explored. Similarly, the allergenic compounds and antinutrients from oilseeds need appropriate processing techniques for innovative safety product development; in this regard, extensive study and research on antinutrients and toxicity are needed. In addition to this, to elevate consumer acceptance of novel food products, studies on sensory, rheological, and physiochemical properties are required. Characterization of oilseed's bioactive compounds is a prerequisite for their implantation as functional ingredients.

## CONFLICT OF INTEREST STATEMENT

The authors declare that they have no conflict of interest.

## ETHICS STATEMENT

The study does not involve any human or animal testing.

## Data Availability

The data of this study are available upon request.
